# YOLO-Ginseng: a detection method for ginseng fruit in natural agricultural environment

**DOI:** 10.3389/fpls.2024.1422460

**Published:** 2024-11-20

**Authors:** Zhedong Xie, Zhuang Yang, Chao Li, Zhen Zhang, Jiazhuo Jiang, Hongyu Guo

**Affiliations:** College of Engineering and Technology, Jilin Agricultural University, Changchun, China

**Keywords:** ginseng fruit, intelligent harvesting, visual detection method, C3f-RN module, model compression

## Abstract

**Introduction:**

The accurate and rapid detection of ginseng fruits in natural environments is crucial for the development of intelligent harvesting equipment for ginseng fruits. Due to the complexity and density of the growth environment of ginseng fruits, some newer visual detection methods currently fail to meet the requirements for accurate and rapid detection of ginseng fruits. Therefore, this study proposes the YOLO-Ginseng detection method.

**Methods:**

Firstly, this detection method innovatively proposes a plug-and-play deep hierarchical perception feature extraction module called C3f-RN, which incorporates a sliding window mechanism. Its unique structure enables the interactive processing of cross-window feature information, expanding the deep perception field of the network while effectively preserving important weight information. This addresses the detection challenges caused by occlusion or overlapping of ginseng fruits, significantly reducing the overall missed detection rate and improving the long-distance detection performance of ginseng fruits; Secondly, in order to maintain the balance between YOLO-Ginseng detection precision and speed, this study employs a mature channel pruning algorithm to compress the model.

**Results:**

The experimental results demonstrate that the compressed YOLO-Ginseng achieves an average precision of 95.6%, which is a 2.4% improvement compared to YOLOv5s and only a 0.2% decrease compared to the uncompressed version. The inference time of the model reaches 7.4ms. The compressed model exhibits reductions of 76.4%, 79.3%, and 74.2% in terms of model weight size, parameter count, and computational load, respectively.

**Discussion:**

Compared to other models, YOLO-Ginseng demonstrates superior overall detection performance. During the model deployment experiments, YOLO-Ginseng successfully performs real-time detection of ginseng fruits on the Jetson Orin Nano computing device, exhibiting good detection results. The average detection speed reaches 24.9 fps. The above results verify the effectiveness and practicability of YOLO-Ginseng, which creates primary conditions for the development of intelligent ginseng fruit picking equipment.

## 1 Introduction

Ginseng, a precious medicinal herb, is a perennial herbaceous plant belonging to the family Araliaceae. It boasts a long history of cultivation and medicinal use and is acclaimed as the “King of Herbs” in China. Ginseng is widely distributed, being cultivated in various regions, including Northeast China, the Korean Peninsula, Japan, Russia, the United States, and Canada (Shuai et al., 2023b; Yoon et al., 2022). Although ginseng varieties differ across locations, their efficacy remains similar. Research indicates that ginseng exhibits various health benefits, such as lowering blood pressure (Chen et al., 2024), protecting the myocardium (Liu et al., 2023), enhancing immune function, and promoting hematopoiesis (Song et al., 2023).

**Figure 1:**
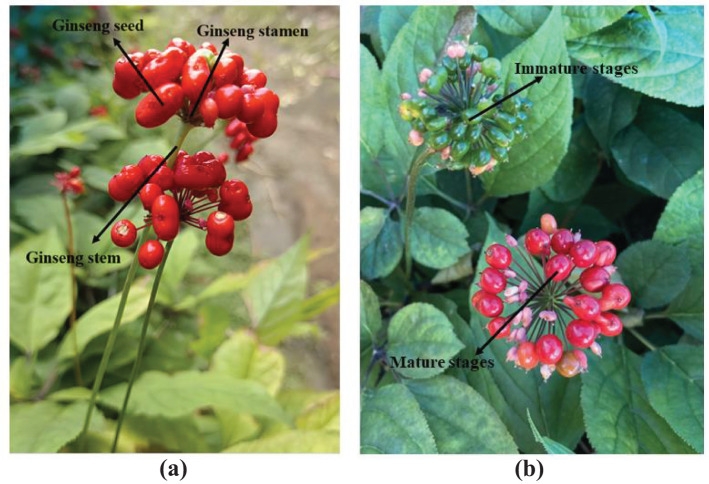
Growth status of ginseng fruit. **(A)** structure of ginseng fruit. **(B)** mature and immature ginseng fruit.

Ginseng fruit, also known as ginseng berries (Rho et al., 2020), is the mature fruit produced by ginseng plants after several years of growth and serves as the seeds of the ginseng plant (Min et al., 2022). The interior of ginseng fruit contains various functional components, including ginsenosides (Liu et al., 2019b; Liu et al., 2022; Wan-Tong et al., 2023), amino acids, proteins, polysaccharides, and Syringaresinol (Choi et al., 2022; Hwang et al., 2023), which have the same medicinal and economic value as ginseng. When ginseng fruit reaches maturity, its surface color transitions from green to a vibrant red, indicating the opportune time for harvesting. The ginseng fruit generally forms at the top of the ginseng plant, with dozens of oval-shaped ginseng seeds densely clustered on the ginseng stamen, arranged in an umbrella-like structure supported by the ginseng stem, as illustrated in **Figure 1A**. The ginseng fruit displays a distinct color difference between the mature and immature stages, as depicted in **Figure 1B**. This study primarily conducts visual detection research on the mature fruits of garden-cultivated ginseng in the Northeast China region. Currently, the harvesting of ginseng fruits has not yet achieved mechanization and automation, still relying on traditional manual harvesting methods. This approach is characterized by low efficiency, high labor strength, and elevated labor costs. Therefore, developing intelligent harvesting equipment for ginseng fruits to replace manual harvesting is a healthy path to promote the sustainable development of the ginseng industry. Building precise and rapid visual detection technology for ginseng fruits is crucial to achieving mechanized harvesting of ginseng fruits, providing essential visual guidance for intelligent harvesting equipment. However, due to the different growth heights of ginseng plants, the growing environment of ginseng fruits is dense and complex, which leads to serious overlap and occlusion problems between ginseng fruits or leaves. Furthermore, due to the unique growth structure of ginseng plants, they are susceptible to disturbances from external environmental factors such as wind direction, resulting in continuous shaking of ginseng fruits or even the lodging of ginseng plants. These circumstances pose challenges for the visual detection task of ginseng fruits. Moreover, the overall growth size of ginseng fruits is relatively small, which also brings a burden to the long-distance viewing of ginseng fruits.

Currently, machine vision technology has been widely applied in the field of agricultural engineering, playing a crucial role in various agricultural tasks such as intelligent harvesting of crops (Shuai et al., 2023a), intelligent monitoring and early warning (Pan et al., 2022), and path navigation in complex field environments (Wang et al., 2022; Luo et al., 2016) proposed a grape automatic detection method for accurately detecting grape clusters in dense environments. This method combines grape color components with the AdaBoost classification framework. It is capable of suppressing the influence of complex background conditions such as weather, tree leaf occlusion, and lighting variations to a certain extent. Liu et al. (2019a) proposed a method for navel orange recognition. Initially, they introduced an improved Otsu threshold segmentation method based on the Cr color component and the centroid filling circle algorithm. This approach effectively identifies the overall contour of navel oranges, leading to a noticeable improvement in recognition accuracy. However, the above method is limited to the target’s geometric shape, color space, surface texture and other characteristic information for detection, it is not suitable for solving the detection problem of ginseng fruit.

With the advancement of machine vision technology, deep learning-based object detection methods have demonstrated significant advantages in terms of accuracy, efficiency, data requirements (Zhang et al., 2024b), generalization capability, and stability (Huang et al., 2023; Zhang et al., 2021). Currently, the one-stage detection method YOLO (You Only Look Once) is rapidly evolving (Terven and Cordova-Esparza, 2023), and its advantages of being lightweight, fast, and accurate enable YOLO to meet the requirements of agricultural operational scenarios. Among them (Li et al., 2022), proposed the tea bud detection method YOLOv3-SPP in order to solve the overlapping and occlusion problems of tea bud detection in dense and complex growth environments. This method introduces SPP (Spatial Pyramid Pooling) (He et al., 2015) into the backbone network of YOLOv3 (Redmon and Farhadi, 2018). Simultaneously, channel and layer pruning algorithms are applied to compress the model. As a result, YOLOv3-SPP achieves a mean average precision of 89.61%, with noticeable reductions in model inference time, weight size, parameter count, and computational cost. (Wang et al., 2023b) replaced the backbone network of YOLOv5 with the inverted residual convolutional modules from the MobileNetv2 (Sandler et al., 2018) network and integrated them with a target association recognition method to design a multi-object selection path. Subsequently, the model’s misjudged output results were corrected using the least squares method, ultimately enhancing the recognition speed and accuracy of the apple harvesting robot effectively (Zhu et al., 2021) introduced a small detection layer into YOLOv5x, combined with Transformer encoder block modules and the CBAM (Convlutional Block Attention Module) (Woo et al., 2018) attention mechanism. This enhancement effectively improves the accuracy of long-distance target detection based on remote sensing images (Ma et al., 2023) proposed the YOLOv5-lotus single-target detection method to detect mature lotus seedpods. In this method, the CA (Coordinate Attention) attention mechanism is introduced at the end of the YOLOv5 backbone network. Ultimately, YOLOv5-lotus achieves an average precision of 98.3% (Yu et al., 2023) proposed a strawberry stolon detection method named Stolon-YOLO. In this method, the authors introduced the HorBlock-decoupled head and Stem Block feature enhancement module into YOLOv7 to facilitate the interaction of high-order spatial information and reduce computational costs. As a result, Stolon-YOLO achieved an average precision of 88.5% for stolon detection, with a computational load of 107.8 GFLOPS (Ang et al., 2024) proposed the YCCB-YOLO detection method for effectively detecting young citrus in dense growth environments. To enhance detection precision while maintaining computational efficiency, this method integrates pointwise convolution (PWonv) lightweight network and simplified spatial pyramid pooling fast-large kernel separated attention (SimSPPF-LSKA) feature pyramid into YOLOv8n. Additionally, the Adam optimization function is utilized to further enhance the model’s nonlinear representation and feature extraction capabilities. As a result, the detection precision of YCCB-YOLO reaches 97.32%.

In conclusion, deep learning-based object detection methods are widely applied in the agricultural domain, however, there is relatively limited research on the detection of ginseng fruits. At the same time, there are overlapping and occlusion problems caused by the intricate growth of ginseng fruits and their leaves in the natural environment, unstable detection problems caused by wind interference from the external environment, and low long-distance detection quality caused by the small overall size of ginseng fruits. The problem also brings difficulties to the task of accurate and rapid detection of ginseng fruits. Therefore, in order to solve the above problems, this study proposed the ginseng fruit detection method YOLO-Ginseng from the perspective of the growth environment and biological characteristics of ginseng fruit. The method first conducts comparative experiments and analyses of several advanced detection methods using a ginseng fruit image dataset. Finally, the YOLOv5s detection method is selected as the base network model, considering its highest model inference speed, minimal computational and parameter requirements, and overall stable detection performance; Next, to enhance the overall detection precision of ginseng fruits, improve the distant detection performance, and enhance the quality of target prediction bounding box localization, a deep-level perceptual feature extraction module named C3f-RN with a sliding window mechanism is designed and integrated into the backbone network of YOLOv5s in a plug-and-play manner. Since the C3f-RN module will reduce the inference speed of the model and increase the model size, this article finally uses a mature channel pruning algorithm to compress the model to make up for the defects brought by the C3f-RN module to the model and strengthen the foundation of model application. In this study, the main contributions are as follows:

1. A new ginseng fruit image dataset was established. The basic data includes 1,519 ginseng fruit images under different angles, scales, light intensity and other conditions.
2. A plug-and-play deep perception feature extraction module C3f-RN with a sliding window mechanism is designed to improve the YOLO-Ginseng backbone network’s feature information extraction capabilities for ginseng fruits and enhance the overall network information transmission efficiency and regression effect.
3. Utilizing channel pruning algorithm to compress YOLO-Ginseng, enhancing model inference speed, and reinforcing model applicability.

## 2 Materials and methods

### 2.1 Image data acquisition

**Figure 2:**
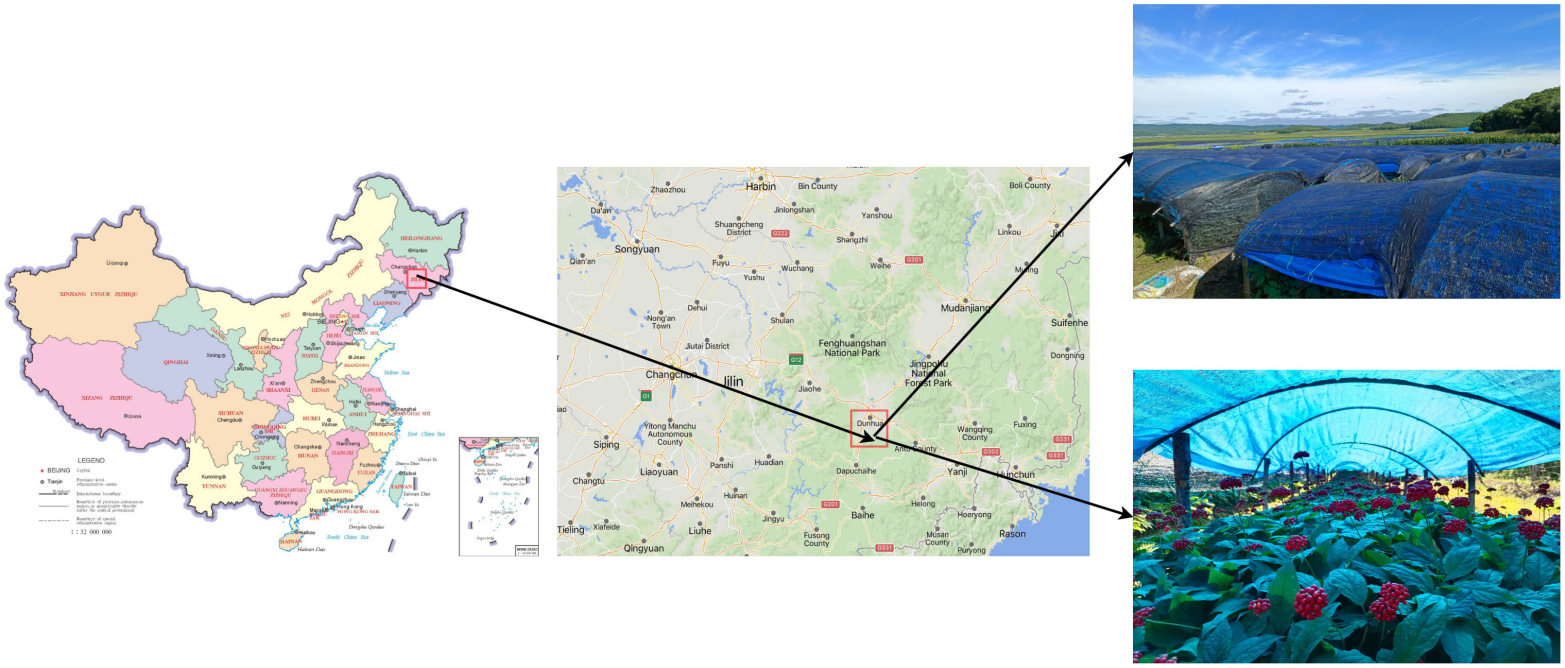
The geographic locations of data collection.

**Figure 3:**
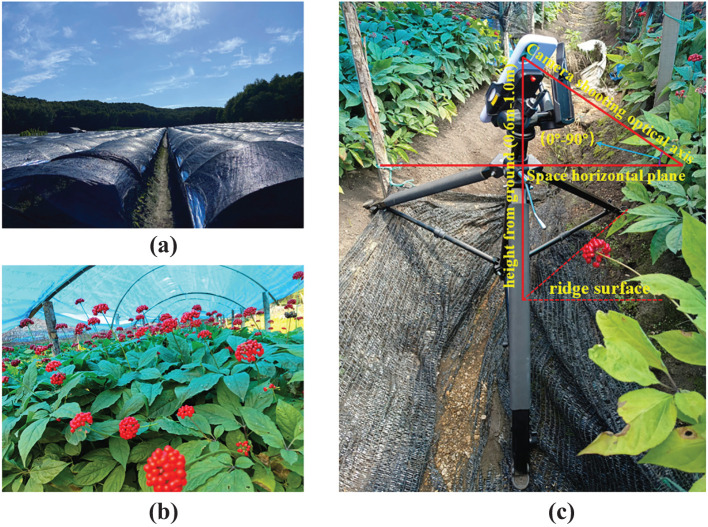
Ginseng planting and image data collection scene. **(A)** The environmental conditions of the ginseng cultivation base. **(B)** The in-field growth conditions of ginseng fruits. **(C)** Data collection platform.

The image data for this study were acquired in August 2023 at the Ginseng Plantation Base in Sandaohezi Village, Dashitou Town, Dunhua City, Jilin Province, China (latitude 43°18’21”, longitude 128°29’41”), as depicted in **Figure 2**. The species collected are ginseng fruits grown from garden ginseng, which is the main ginseng species grown in Northeast China. The environmental conditions of the ginseng cultivation base are illustrated in **Figure 3A**. The ginseng is grown using a wide-ridge shed planting pattern, with a ridge width of approximately 1.60m-1.70m and a ridge height above the ground of approximately 0.16m-0.20m. **Figure 3B** illustrates the in-field growth conditions of ginseng fruits, with ginseng plants spaced approximately 0.10-0.15m apart and exhibiting varying growth heights ranging from approximately 0.30m to 0.60m. The ginseng fruits grow densely and chaotically, displaying significant disparities in growth height. In summary, ginseng fruit grows in a complex environment, with high density and chaotic distribution, and the landform environment is relatively poor. Despite the protection of the trellis, ginseng fruit is still affected by the intensity of external light. Therefore, in order to accurately collect various types of image data of ginseng fruit in a complex environment and effectively restore its growth state, this puts forward certain requirements for the data acquisition method. It is necessary to try to avoid collecting image data with unclear characteristic information of ginseng fruit, such as image data with overexposure of brightness and blurred pixels.

Given the high standards of ginseng cultivation and the complex growth environment of ginseng fruits, in order to effectively enhance the quality of ginseng fruit detection and obtain reliable experimental data, facilitating subsequent research on the efficiency of automatic detection and operational convenience of intelligent ginseng fruit picking equipment, the methods for ginseng fruit image data collection in this study are as follows: As shown in **Figure 3C**, Data collection work was conducted using the wide-angle camera of an iPhone 11 smartphone. Initially, the image storage format was set to JPEG with a resolution of 4032×3024 or 3024×4032. HDR mode was activated to enhance exposure details, and automatic focus and exposure modes were selected. Turn on the anti-shake function. Start the 3-second delayed shooting or continuous shooting mode when acquiring image data required by special scenes; Secondly, the mobile phone was fixed on the camera stand and adjusted manually within a range of about 0.6m-1.0m from the ground. The rotating joint of the camera stand was adjusted reasonably to ensure that the angle of the optical axis of the mobile phone relative to the horizontal plane of the space was within the range of 0°-90°for data collection. Among them, according to the growth characteristics of ginseng fruit and the needs of mechanical equipment, this study obtained global image data of ginseng fruit at different angles, different scales and different background conditions at shooting angles of 0°, 45° and 90°. In order to ensure the randomness of image data and increase the diversity of data samples, this study also collected image data from other shooting angles. In order to restore the growth conditions and surrounding environment of ginseng fruit as closely as possible, the image data collection work also utilized the data collection method of manual handheld shooting, and also carried out data collection work according to the light intensity conditions in different time periods. Finally, a total of 1664 pieces of image data were collected. After cleaning some of the image data with unclear ginseng fruit characteristic information, 1519 pieces of image data remained.

### 2.2 Data augmentation and establishment of a ginseng fruit image dataset

In order to enhance the quality of the image dataset and achieve more equitable detection results, this study strictly adheres to annotation principles for annotating the image data. Single-object annotation was performed using LabelImg 1.8.6, designated as “RSZ,” with label files in txt format. It is noteworthy that ginseng fruits posing difficulty for human eye discernment or having occlusion areas greater than 90% were excluded from annotation. Finally, the annotated image data were randomly partitioned according to a 7:2:1 ratio, yielding 1063 images for the training set, 304 images for the validation set, and 152 images for the test set.

**Figure 4:**
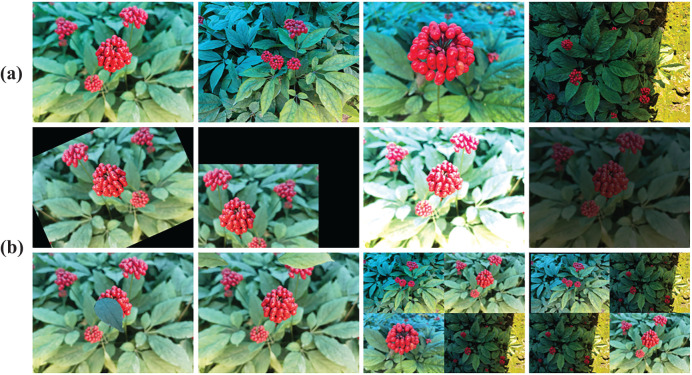
Example of image data augmentation. **(A)** Example of original image data. **(B)** Example of augmentation techniques, including random rotation, random translation, brightness adjustment, simulated occlusion, and data concatenation, sequentially.

Due to the complex growth environment of ginseng fruits, the manually collected data samples cannot effectively replicate the distinctive characteristics of the ginseng fruit’s growth environment. To ensure the integrity and diversity of the data samples and improve the generalization ability of the network model, this study employed data augmentation techniques such as affine transformation, simulated occlusion, and data concatenation on the training set. Through a random combination approach, the dataset was expanded, subsequently, manual means are used to check and delete the incorrectly labeled noise data to ensure the correctness of the expanded data (Zhang et al., 2024a), ultimately yielding 2415 images in the training set. It is worth noting that the image data in all training sets, validation sets, and test sets are distinct from each other. An example of augmented image data is illustrated in **Figure 4**, and detailed information on the ginseng fruit image dataset is provided in **Table 1**.

**Table 1:** Detailed information on the ginseng fruit image dataset.

Dataset	Raw data	Augment data	Training set	Validation set	Test set
**RSZ**	**1519**	**1352**	**2415**	**304**	**152**

The bold values indicate the number of image data in each stage dataset.

### 2.3 YOLO-Ginseng

**Figure 5:**
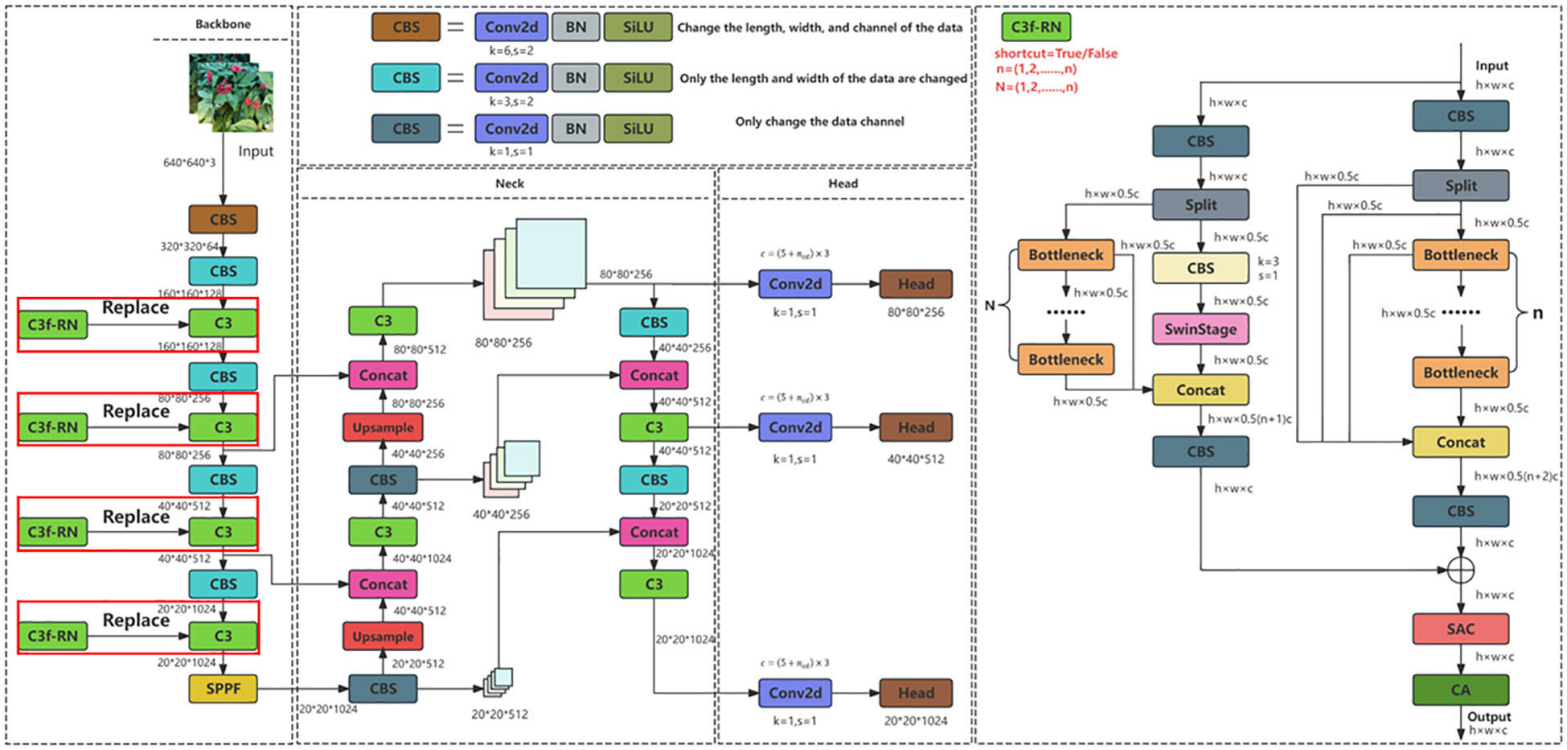
YOLO-Ginseng network structure.

In fact, during the real-time detection process of ginseng fruits, in order to avoid the interference of external environmental changes on ginseng fruit detection and improve the detection effect of ginseng fruit in complex environments, the detection method is required to have a higher model reasoning speed and excellent image data processing capabilities. In other words, the detection network can quickly and accurately obtain and process the global image feature information of ginseng fruit when performing detection tasks. Therefore, for the mechanized harvesting task of ginseng fruits in complex agricultural environments, the ability to be deployed on edge computing devices and possess high detection speed is the primary consideration in this study, this forms an important foundation for real-time detection of ginseng fruits. Secondly, consideration is given to how to improve the overall detection quality of ginseng fruits. Based on the above analysis, this study first conducted preliminary experimental analyses of advanced and typical detection methods, namely YOLOv5s (Jocher et al., 2020), YOLOv7 (Wang et al., 2023a), YOLOv8s (Jocher et al., 2023), and YOLOv9-C (Wang et al., 2024), considering factors such as model inference speed, computational cost, weight size, and detection accuracy. The results show that YOLOv5s has the fastest inference speed and the lowest computational cost and weight size. Although YOLOv5s performs weakly in average precision, its detection effect on ginseng fruits in complex scenes is excellent, which shows that relying solely on average precision cannot fully measure the quality of the model. Given the advantages of YOLOv5s in speed and resource consumption, and its precision has reached a high level, this study finally selected YOLOv5s as the basic network model for ginseng fruit detection research, and finally the average precision of ginseng fruit detection will be improved through technical means. efforts will be made to improve the average precision of ginseng fruit detection through technical means. The YOLOv5s network architecture mainly consists of four parts: the input module, the backbone network, the neck network, and the head network. The input module is used to preprocess the input image data, including data augmentation, adaptive resizing, and adaptive anchor box calculation; The backbone network adopts the CSPDarknet53 structure to extract feature information from the input image data; The neck network utilizes the FPN (Feature Pyramid Network) structure and the PAN (Pyramid Attention Network) structure to integrate the extracted feature information; The head network is responsible for performing simple object classification, position regression, and confidence inference predictions on the final feature information, thereby generating the ultimate detection results. Based on the preliminary experimental analyses, it was found that YOLOv5s is still insufficient to handle the visual detection tasks of ginseng fruits. Therefore, in order to improve the average precision of YOLOv5s in detecting ginseng fruits, enhance the long-distance detection effect of ginseng fruits and the quality of target prediction bounding box positioning, and solve the problem of detection difficulties caused by occlusion or overlap of ginseng fruits and interference from the external environment, this study finally proposes a detection method YOLO-Ginseng (Ginseng, You Only Look Once). In this detection method, the deep-level perceptual feature extraction module C3f-RN, designed in this study with a plug-and-play sliding window mechanism, is integrated into the backbone network of YOLOv5s. Finally, a channel pruning algorithm is employed to compress the model, compensating for the shortcomings introduced by C3f-RN and enhancing the model’s applicability. The model network architecture of YOLO-Ginseng is illustrated in **Figure 5**.

#### 2.3.1 C3f-RN

**Figure 6:**
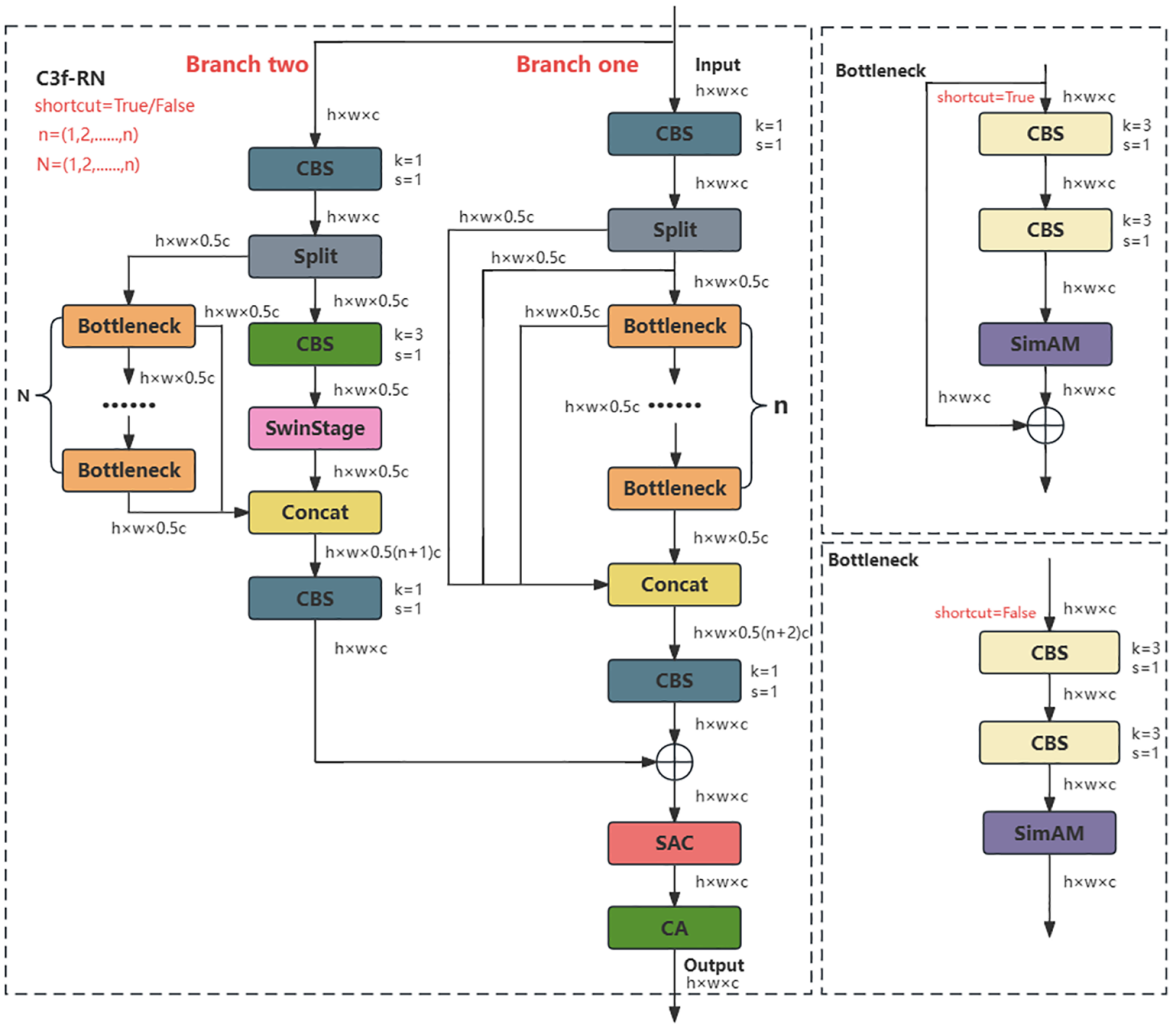
C3f-RN network structure.

**Figure 7:**
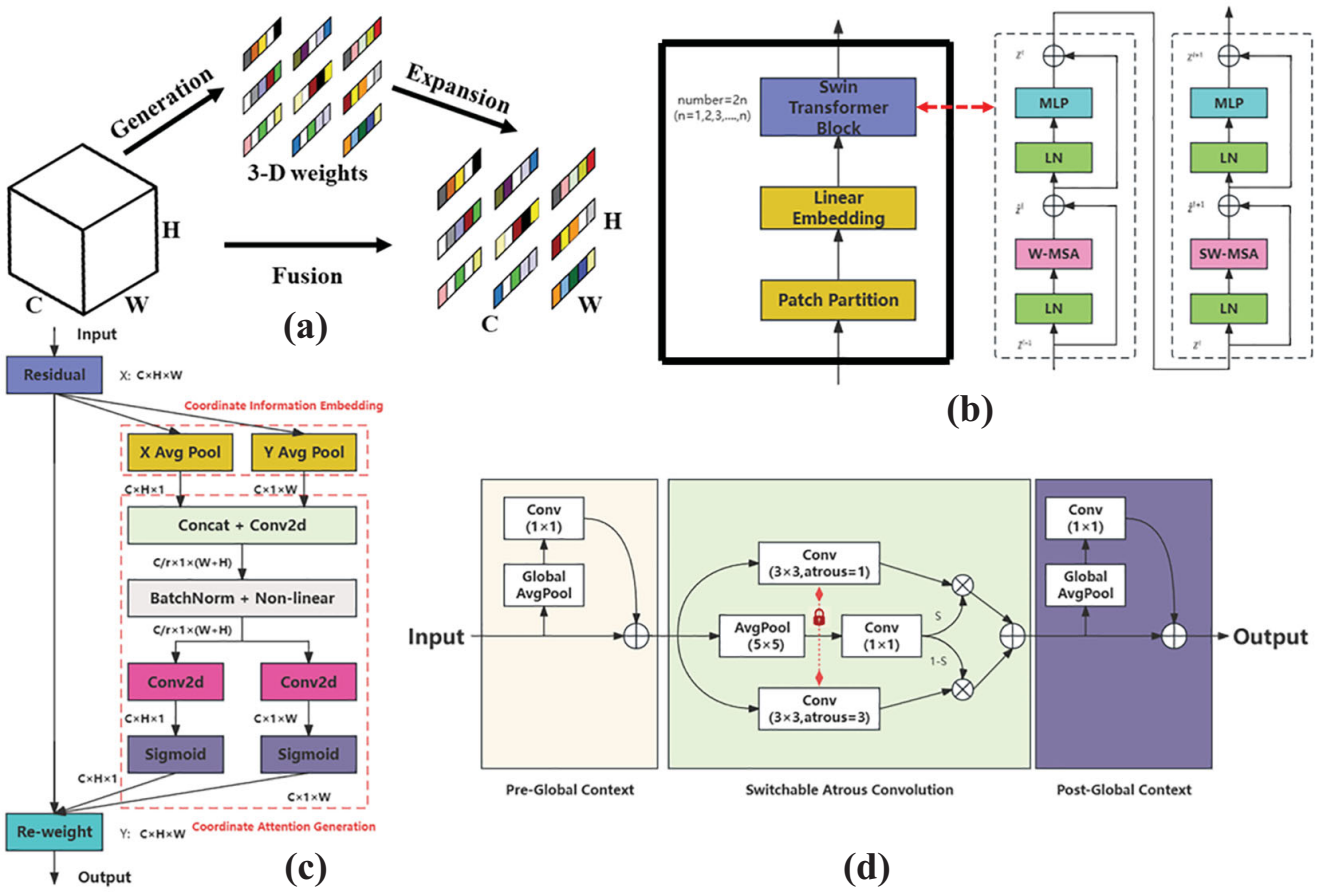
**(A)** Structure of the SimAM. **(B)** New swin stage structure. **(C)** Structure of the CA. **(D)** Structure of the SAC.

Due to the unique biological structure and growth environment of ginseng fruits, their mature bright red color is easily distinguishable from the background. From a two-dimensional pixel image perspective, the detection of ginseng fruits can be regarded as a binary classification problem, where each pixel is classified into two categories: red representing ginseng fruits and green representing the background. Therefore, this study aims to enhance the feature extraction capability of the YOLOv5s backbone network, enabling it to accurately and effectively process or distinguish these binary pixel points to improve the detection performance of ginseng fruits. As is well-known, deepening and widening the overall hierarchical structure of a network can potentially expand the network’s receptive field and enhance its learning capability. Therefore, this study adopts the design principles of the residual network ResNet (He et al., 2016) and combines them with the advantages of the C3 structure to design a plug-and-play deep-level perceptual feature extraction module named C3f-RN, which incorporates a sliding window mechanism, as illustrated in **Figure 6**. C3f-RN possesses a unique network structure and introduces the novel Swin Stage module along with the SAC (Switchable Atrous Convolution) convolution (Qiao et al., 2021). It effectively combines with lightweight attention mechanisms, including CA (Coordinate Attention) (Hou et al., 2021) and a simple, parameter-free attention module called SimAM (A Simple, Parameter-Free Attention Module) (Yang et al., 2021), to assist in enhancing the ability of the C3f-RN module to extract both global and detailed features of ginseng fruits. This ultimately achieves the advantages of plug-and-play functionality. The overall workflow and advantages of the C3f-RN module are as follows:

(1) First, the initial characteristic information of ginseng fruits is input and allocated to two main branches, namely branch one and branch two. Among them, multiple Bottlenecks are introduced on two branches respectively in a direct and residual manner, deepening the hierarchical structure of the network and helping C3f-RN to extract ginseng fruit image feature information in more detail. Simultaneously, in both branches, Split operations are applied to set hidden channel branches in the channel dimension and extend the unique feature processing operations of the Swin Stage. This design not only widens the network structure and potentially expands C3f-RN’s field of vision for ginseng fruit feature information, but also increases the diversity of C3f-RN’s processing of ginseng fruit feature information and enriches the flexibility of the network structure. It is worth noting that the combination of branch one and branch two ultimately forms a unique C3 structure.

(2) On branch one, the initial feature information of ginseng fruit is first subjected to a 1×1 convolution for dimensionality reduction, adjusting the number of feature information channels while helping the network learn more complex feature information; Next, the obtained feature information is halved and separated in the channel dimension through Split operation, with one part directly outputted through the residual branch and the other part outputted through n Bottleneck units. A series of Bottleneck units are responsible for conducting certain feature extraction operations on the input feature information through two 3×3 convolutions, enabling the network to learn more, finer, more abstract, and advanced feature information; Finally, the output parts of the two are concated in the channel dimension and undergo a 1×1 convolution again to transform the fused feature information in the channel dimension to facilitate the forward propagation of the fused feature information. Among them, the Bottleneck structure with residual branches is used on branch one, and the SimAM attention module is introduced at the end of the structure to further improve the network’s attention to and retention of the detailed information of the ginseng fruit when extracting feature information in each small step. The SimAM attention module introduces the concept of three-dimensional attention for the first time, as illustrated in **Figure 7A**. Based on neuroscience theory, SimAM calculates the importance of each neuron by optimizing the energy function to adjust the attention weight distribution shape. The advantage of this module is that there is no need to add redundant parameters to the original network, and the three-dimensional attention weight information of the feature map can be inferred using a small number of parameters. Therefore, based on its advantages, this study embeds the SimAM attention module at the end of each Bottleneck structure in the network, without affecting the overall parameter count of the C3f-RN module. This enables each Bottleneck to automatically learn and dynamically adjust attention weight distribution ratios based on the input feature information of different categories. Consequently, it guides each Bottleneck to focus more on the detailed feature information of ginseng fruits and filter out irrelevant feature information.

(3) On branch two, the initial feature information of ginseng fruit is still subjected to a 1×1 convolution operation; Secondly, the obtained feature information is halved and separated in the channel dimension through the Split operation. One part undergoes N Bottleneck output through the residual branch, and the other part undergoes a 3×3 convolution for feature extraction and is directly input to the Swin Stage module for further processing. Processed in one step. Part of the Bottleneck here has a residual structure, and part of it has no residual structure and is set to a interleaved arrangement. This unique design arrangement is to make the network more flexible when processing feature information, improve the generalization ability of the network, and enhance embeddability of C3f-RN modules.

The Swin Stage module is a novel structural design based on the most crucial component of the Swin Transformer model (Liu et al., 2021). It extracts the primary stage of the Swin Transformer model, encapsulating the Patch Partition operation and Linear Embedding operation within the same stage while eliminating the Patch Merging operation. The overall structure of the Swin Stage module is illustrated in **Figure 7B**. The module first divides the input ginseng fruit feature information into multiple fixed-size and non-overlapping local feature map blocks through Patch Partition operation, enabling independent processing of each block. This aids in better capturing local feature details; Next, through the Linear Embedding operation, the input feature information is mapped to a lower-dimensional feature space to reduce feature dimensionality and obtain more compact feature information. Finally, the feature information processed by the upper layer is input to the even-numbered stacked core unit Swin Transformer Block for information transformation and interactive operations to achieve the extraction and integration of feature information. The unique Swin Stage structure enables the C3f-RN module to rapidly capture the global feature information of ginseng fruits. Its efficient cross-window information interaction processing capability allows the C3f-RN module to constantly grasp and learn the detailed information of ginseng fruits at different pixel positions, enhancing sensitivity to this information. This effectively addresses detection challenges arising from overlap or occlusion between ginseng fruits or leaves, thereby assisting and improving the quality of target prediction bounding box positioning; Finally, the outputs of both branches are concatenated along the channel dimension and then passed through another 1×1 convolutional layer to transform the fused feature information in the channel dimension, facilitating the propagation of the fused feature information forward.

(4) The processed feature information from branches one and two are separately passed through 1×1 convolutions and then summed up. Subsequently, SAC convolutional operations are employed to assist the C3f-RN module in further enhancing the overall network’s global receptive field for ginseng fruit, ensuring the integrity of information weights, and improving the expressive capability of the C3f-RN module. Among them, SAC consists of Atrous Convolution, Pre- and Post-Context Modules, and is combined with a Switch Mechanism. This module adjusts the dilation rate and switch value of Atrous Convolution to perceive details of targets at different scales, reducing information loss and enhancing the model’s ability to process image data. Its structure is illustrated in **Figure 7D**. The incorporation of SAC effectively enhances the capability of C3f-RN in handling multi-scale ginseng fruit detection, assisting the backbone network in capturing feature information of distant ginseng fruits. Finally, this study quoted the CA attention mechanism at the end of the C3f-RN module structure to help the C3f-RN module integrate and select the captured and processed ginseng fruit feature information in the final stage, and eliminate redundant information. The remaining information is directly output to the next layer of network, with the ultimate goal of protecting the characteristic information of ginseng fruits. Among them, the CA attention mechanism is a lightweight attention mechanism that enhances feature representation capabilities. Its core idea is to embed positional information in the feature channel dimension, decomposing channel attention into aggregated features along two spatial directions. One direction captures long-range dependencies, while the other preserves precise positional information. Finally, the two are complementarily fused to learn the importance weights of different channels, thereby enhancing interest in complex target features and suppressing redundant or noisy channels. Its structure is illustrated in **Figure 7C**.

The C3f-RN module, with its flexible and unique network structure, not only deepens and widens the hierarchical structure of the network but also effectively integrates other advanced feature information processing models. Furthermore, the input feature information can be maintained unchanged in size through a series of 1×1 convolutions, Concat, and addition operations, enabling the C3f-RN module to be easily integrated into other networks, achieving the advantage of plug-and-play. However, although the designed C3f-RN module in this study can effectively extract the features of ginseng fruits in complex backgrounds and enhance the overall detection performance of ginseng fruits, its integration into the backbone network of YOLOv5s increases the parameter count and computational burden of the overall YOLO-Ginseng network model, resulting in a reduction in the model’s inference speed, as shown in **Table 2**. Therefore, this study aims to address this issue by employing mature channel pruning algorithms to mitigate the drawbacks introduced by the C3f-RN module to the overall YOLO-Ginseng network model.

**Table 2:** Comparing C3f-RN structural parameters.

Network +C3f-RN	depth	n heads	win size	$AP_{0.5}$ (%)	T (ms)	Size (MB)	parameters	GFLOPS
**None**	**-**	**-**	**-**	**93.2**	**5.5**	**14.4**	**7022326**	**15.9**
**(1)**	**2**	**8**	**1×1**	**94.2**	**21.3**	**44.5**	**21990962**	**36.0**
**(2)**	**2**	**4**	**1×1**	**94.2**	**21.3**	**44.5**	**21990930**	**36.0**
**(3)**	**2**	**2**	**1×1**	**93.9**	**21.3**	**44.5**	**21990914**	**36.0**
**(4)**	**2**	**1**	**1×1**	**93.5**	**21.3**	**44.2**	**21990906**	**35.9**
**(5)**	**2**	**16**	**4×4**	**94.5**	**21.3**	**44.8**	**21997170**	**36.1**
**(6)**	**2**	**8**	**4×4**	**95.8**	**21.3**	**44.5**	**21994034**	**36.0**
**(7)**	**2**	**4**	**4×4**	**94.3**	**21.3**	**44.5**	**21992466**	**36.0**
**(8)**	**2**	**2**	**4×4**	**94.0**	**22.3**	**44.5**	**21991682**	**36.0**
**(9)**	**2**	**1**	**4×4**	**93.9**	**21.3**	**44.3**	**21991290**	**35.9**
**(10)**	**2**	**8**	**7×7**	**95.3**	**22.4**	**45.2**	**22991682**	**36.3**
**(11)**	**2**	**4**	**7×7**	**94.4**	**21.3**	**45.0**	**21996306**	**36.2**
**(12)**	**2**	**2**	**7×7**	**94.2**	**21.3**	**44.9**	**21993602**	**36.1**
**(13)**	**2**	**1**	**7×7**	**93.7**	**21.3**	**44.8**	**21992250**	**36.1**

The bold value indicates that each set of experiments under different parameter configurations shows the best results under different evaluation indicators.

#### 2.3.2 Model compression

To effectively reduce the parameter count and computational burden of the YOLO-Ginseng overall network model, enhance model inference speed, and preserve the significant effects of the C3f-RN module on detecting ginseng fruits, this study utilizes mature channel pruning algorithms (Liu et al., 2017) to compress the YOLO-Ginseng overall network model. The channel pruning algorithm mainly consists of three steps: sparse training, model pruning, and model fine-tuning.

**Figure 8:**
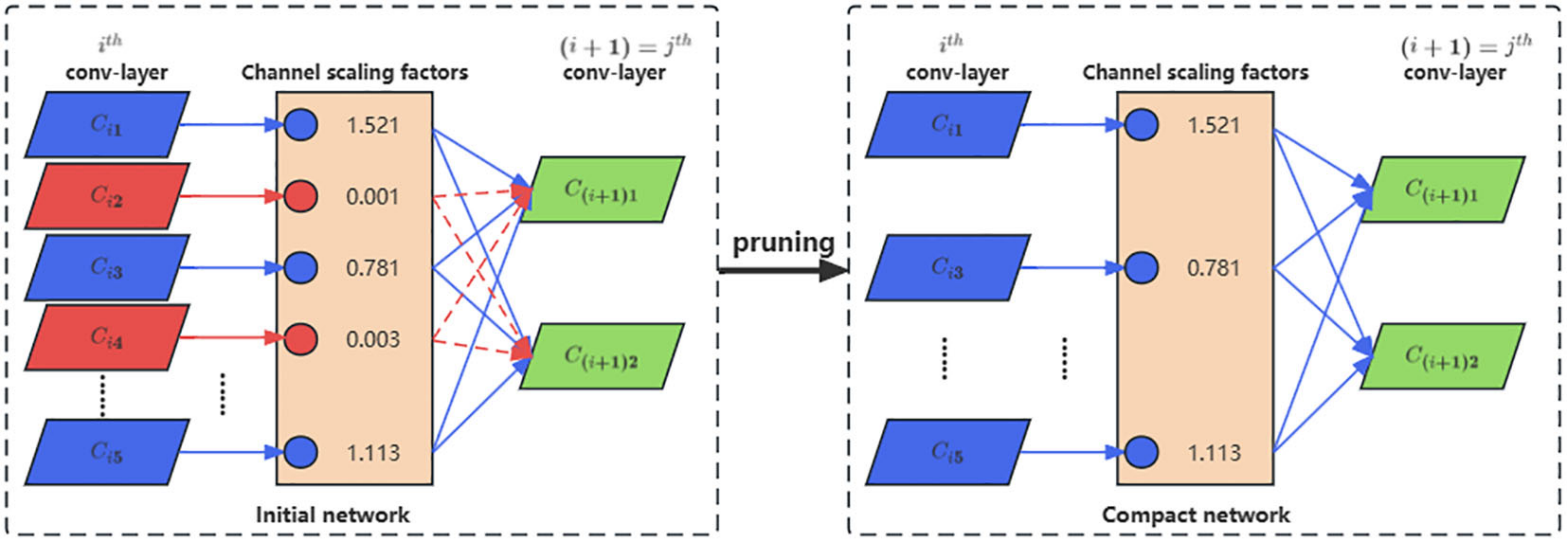
The principle of model channel pruning, where the blue portion signifies channels with high contribution, and the red portion indicates channels with low contribution.

Firstly, sparse training is the initial step in model compression, representing a crucial technique for reducing the model parameter count to optimize both the training and inference processes. This study employs the L1 norm method and iteratively adjusts the sparsity parameter λ multiple times to strike a balance between the model’s sparsity and accuracy. This process ensures that the scaling factor γ coefficient of the BN (Batch Normalization) layer converges rapidly to zero. The variation in the γ coefficient adjusts the range and degree of change in the BN layer’s output feature information, thereby influencing the changes in BN layer parameter weights and the subsequent learning capacity of the model. Ultimately, it reveals the contribution levels of each channel to the network computations. Specifically, for the input x, weights W, scaling factor γ coefficient, and bias term β of the BN layer, the calculation of the BN layer output y is shown in Equation 1:


(1) $$\text{y}=\text{γ}\left(\frac{\text{x}-\text{μ}}{\sqrt{{\text{σ}}^{2}+\text{ϵ}}}\right)+\text{β}$$

where μ denotes the input mean, σ represents the input standard deviation, ϵ is a small constant for numerical stability, and γ and β are learnable parameters. Secondly, model pruning is performed, as illustrated in **Figure 8**. The basic principle involves utilizing the γ coefficients of BN layers based on the sparse training results to assess the contribution levels of channels to the network. High-contribution channels are retained, while low-contribution channels are pruned, ultimately consolidating the deep network structure to compress the model. Finally, due to the sparse training and pruning of the model, which lead to a decrease in detection accuracy, the model requires fine-tuning. Model fine-tuning involves readjusting the pruning weights, using the compressed model as a pre-trained model for further iterative training until the detection performance of the model is restored.

### 2.4 Evaluation standard

This study employs seven model evaluation standard to assess the detection performance of YOLO-Ginseng. The evaluation standard consist of precision (P), recall (R), average precision (AP), model weight size (MB), model detection efficiency (evaluated through the model inference time on the validation set (ms), including image data preprocessing time (pre-process), network model inference time (inference), and non-maximum suppression time for processing predicted targets (NMS-per)), model parameter count (parameters), and model computational load (GFLOPS). Where P represents the proportion of the actual positive samples during the model detection process to the total predicted positive samples by the model. R is the proportion of the predicted actual positive samples by the model to the total actual positive samples. AP is the area under the PR curve formed by P and R. The calculations are shown in Equations 2-4:


(2) $$\text{P}=\frac{\text{TP}}{\text{TP}+\text{FP}}$$

(3) $$\text{R}=\frac{\text{TP}}{\text{TP}+\text{FN}}$$

(4) $$\text{AP}=\int_{0}^{1}\text{P}\left(\text{R}\right)\text{dR}$$

where TP is the count of samples predicted as positive and are actually positive; FP is the count of samples predicted as positive but are actually negative; FN is the count of samples predicted as negative but are actually positive. The confidence IoU is the overlap ratio between the predicted bounding box and the true bounding box, with a threshold typically set at 0.5. When the network-computed overlap ratio IoU is greater than 0.5, the sample is considered TP; otherwise, the sample is considered FP. This study takes into account the application requirements of YOLO-Ginseng in actual agricultural scenarios, and uses $AP_{0.5}$ (IoU=0.5) as the average accuracy index of YOLO-Ginseng for ginseng fruit detection.

## 3 Results

### 3.1 Experimental setting

This study will evaluate, compare, and discuss YOLO-Ginseng through multiple sets of experiments to verify its effectiveness, stability, and practicality in performing ginseng fruit detection tasks. It is noteworthy that all experiments in this study were conducted on the same PC device and based on the PyTorch framework for training, validation, and testing. The specific experimental environment is presented in **Table 3**. YOLO-Ginseng will employ transfer learning to enhance training speed. Where the number of epochs is set to 200, batch size is 8, and the image input size is 640×640. The experiment utilized the Stochastic Gradient Descent (SGD) optimizer, with the remaining parts configured with the parameters from hyp.scratch-low.yaml in the official YOLOv5-7.0 version by default.

**Table 3:** Experimental environment configuration.

Configuration name	Environmental and version
**System**	**Windows**
**CPU**	**12th Gen Intel(R) Core (TM) i7-12700H**
**GPU**	**NVIDIA GTX3060**
**Running memory**	**32G**
**Graphics card memory.**	**6G**
**CUDA**	**11.7**
**Python**	**3.10.9**
**Pytorch**	**2.0.0**

The bold values indicate the environment configuration, version, and parameters used in each experiment.

### 3.2 The overall detection performance of YOLO-Ginseng

**Figure 9:**
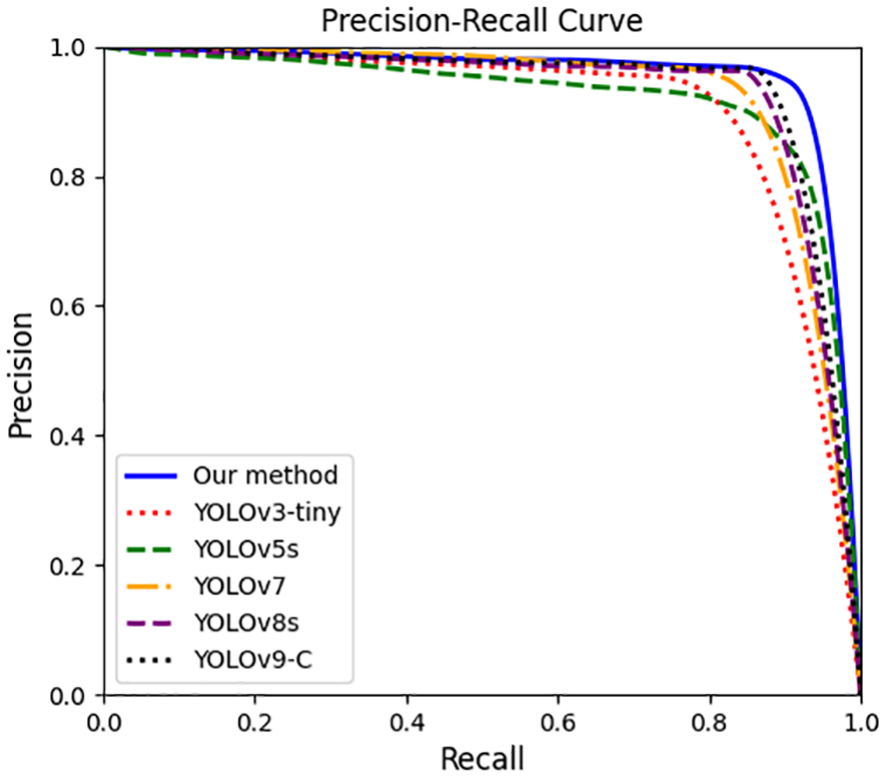
PR curves of different models.

**Figure 10:**
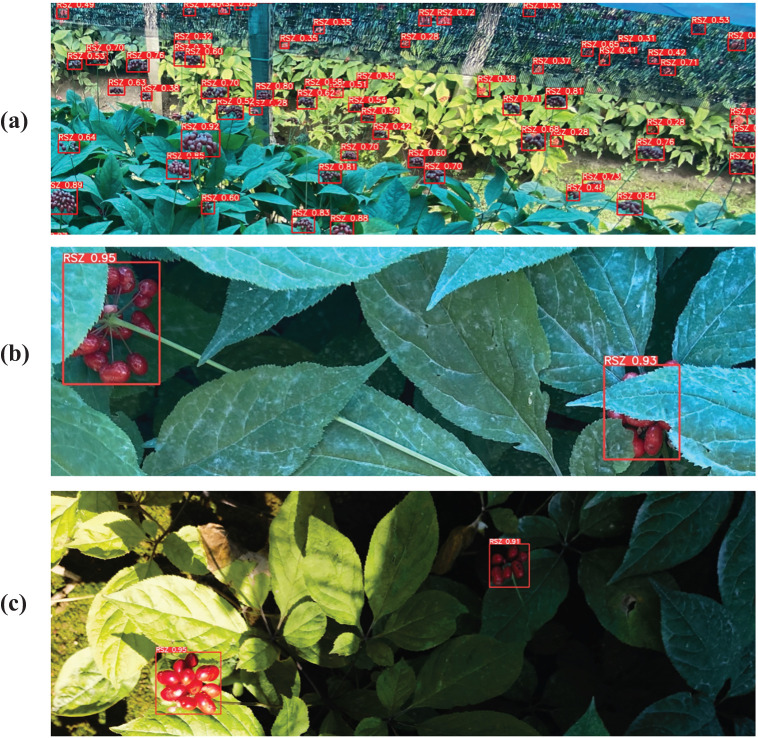
Detection performance of YOLO-Ginseng on ginseng fruits in different scenarios. **(A)** Dense and complex scenarios. **(B)** Scene with occlusions. **(C)** Scenes with varying light intensity.

The YOLO-Ginseng proposed in this study, after training, validation, testing, and compression, demonstrates specific evaluation results and detection performance are shown in **Table 4**; **Figures 9**, **10**. The results indicate that YOLO-Ginseng achieves precision, recall, and average precision of 93.6%, 91.1%, and 95.6%, respectively. The model inference time is 7.4 ms, with parameters totaling 4,545,903, computational cost reaching 9.3 GFLOPS, and the final model weight size being 10.5 MB. As can be seen from the blue PR curve in **Figure 9**, YOLO-Ginseng demonstrates excellent average precision performance when trained with pre-trained weights (yolov5s.pt). As shown in **Figure 10**, YOLO-Ginseng maintains high detection quality in various scenarios, including densely complex scenes in images (a), overlapping and occluded situations in images (b), and varying light intensity conditions in images (c). It exhibits broad detection coverage and accurate localization of object prediction bounding boxes. However, in the dense and complex scene in images (a), the regression confidence of long-distance detection of tiny ginseng fruits is low. This may be due to the fact that the long-distance ginseng fruit targets are small and are obscured by other environmental factors or their own fruits during detection. In summary, YOLO-Ginseng performs excellently in the single-target detection of ginseng fruits in agricultural natural environments.

**Table 4:** Evaluation results of YOLO-Ginseng.

Network	P (%)	R (%)	$\;\;\;\;AP_{0.5}\;$ (%)	T (ms)	Size (MB)	Parameters	GFLOPS
**YOLO-Ginseng**	**93.6**	**91.1**	**95.6**	**7.4**	**10.5**	**4545903**	**9.3**

The bold values indicate the best results of “YOLO-Ginseng” on each evaluation metric.Here I would like to remind you that the value corresponding to Parameters should be changed to 4545903.

### 3.3 Study on the impact of optional parameters in C3f-RN

Given the diversity of content and complexity of the structure in the C3f-RN module, it is essential to investigate its impact on YOLO-Ginseng. Due to the effective integration of the novel Swin Stage module within the C3f-RN module, it is necessary to investigate the impact of the Swin Transformer Block’s quantity (depth), the number of attention heads (n heads), and the size of the local window (win size) in the Swin Stage module on the model. This study will conduct a comparative test analysis based on the official setting experience and training configuration performance of these three parameters and based on the ginseng fruit image data set to determine the best comprehensive parameter value. The parameter comparison results are shown in **Table 2**. The results demonstrate that the integration of the C3f-RN module with the YOLOv5s backbone enhances the average precision of YOLO-Ginseng. However, this fusion increases the model’s size, parameter count, and computational load, thereby extending the model’s inference time. Among them, test (6) raised the average precision of the model to the highest value, which was 2.6% higher than YOLOv5s in $\;AP_{0.5}$ . Therefore, this study finally selected the data in experiment (6) as the final structural parameters of the C3f-RN module: depth is 2, n heads is 8, and win size is 4×4.

The rules for the above parameter settings are as follows: To simplify the model structure and improve computational efficiency, the C3f-RN module primarily adopts the first stage of the Swin Transformer in the Swin Stage module, eliminating the Patch Merging operation; therefore, the parameter depth is set to 2. Secondly, the value of n heads will be reasonably determined based on win size and training configuration performance, as calculated by the following Equations 5, 6:


(5) $$\text{num}\;\text{wins}=\frac{\text{H}\times \text{W}}{\text{win}\;\text{size}\times \text{win}\;\text{size}}$$

(6) $$\text{n}\;\text{heads}=\sqrt{\text{num}\;\text{wins}}$$

Where num wins represents the number of windows into which the input feature information is divided, and H and W represent the height and width of the input feature information, respectively. In this study, H and W are set to the average size of the feature information input into the C3f-RN module from the backbone network. The win size controls the range of the local attention mechanism, where a larger win size helps the model learn longer dependencies but increases computation and memory costs. On the other hand, a smaller win size aids in introducing a local attention mechanism, but excessively small window sizes may limit the model’s ability to capture global dependencies. Therefore, in accordance with the empirical settings provided by the official documentation, this study conducted comparative experiments with three different sizes of local windows: 1×1, 4×4, and 7×7. In principle, the value of n heads should ideally be equal to num wins. However, to reduce the computational cost of the model, enhance feature extraction efficiency, and consider overall training configuration performance, this study sets the value of n heads to be the square root of num wins, thus obtaining a reasonable range of values. Secondly, determine a certain integer through the value range and at the same time take an even multiple of the integer upward until it is 1. Finally, experimental results are used to eliminate outliers, and the remaining values are considered as the appropriate n heads (The calculation formula is derived from multiple comparative experiments and manual parameter adjustment).

### 3.4 YOLO-Ginseng compression performance

**Figure 11:**
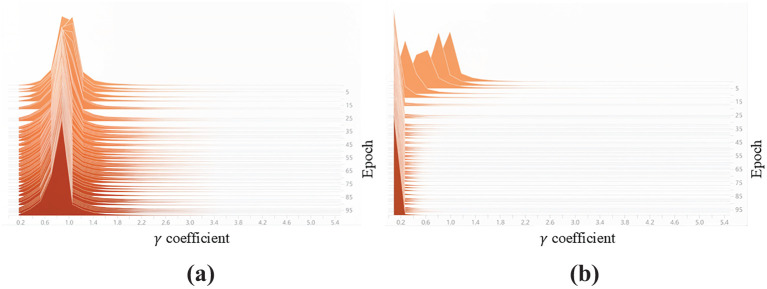
Distribution of the γ coefficient in the BN layer. **(a)** Before sparse training. **(b)** After sparse training.

According to the channel pruning algorithm steps, the first step is to determine an appropriate sparsity rate  λ  for conducting sparse training on the model. The comparative results of training with different sparsity rates are shown in **Table 5**. The results indicate that when selecting a sparsity rat λ  of 0.002, the average precision of YOLO-Ginseng decreases to 91.6%. **Figure 11** shows the γ coefficient distribution form of the BN layer. It can be seen that the γ coefficient distribution center gradually and rapidly converges to 0, and becomes stable after 100 rounds of iterative training. In conclusion, selecting a sparsity rate λ  of 0.002 for sparse training is deemed more reasonable for the model.

**Table 5:** Comparison of training performance under different sparsity rate.

Spares rate ( λ )	$AP_{0.5}$ (%)
**0**	**95.8**
**0.001**	**91.3**
**0.002**	**91.6**
**0.003**	**84.1**
**0.004**	**82.3**
**0.005**	**78.9**

The bold values indicate the best results achieved by the model at different sparsity rates.

**Figure 12:**
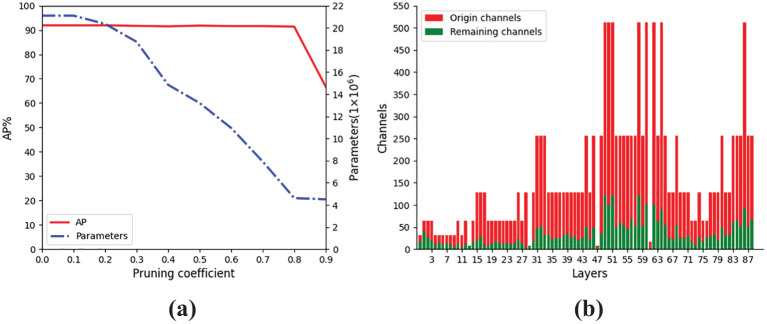
Channel pruning effects of the model. **(A)** Effects of different pruning coefficient on the model. **(B)** Changes in the number of channels for each layer before and after pruning.

After the model is sparsely trained, it is necessary to determine the appropriate pruning coefficient r to prune channels with low model contribution. This study uses 0.1 steps to select appropriate pruning coefficients, the model pruning changes are shown in **Figure 12**. The results show that after the pruning coefficient is 0.8, the average accuracy of YOLO-Ginseng decreases, while the model parameters change slightly, which shows that when the pruning coefficient is 0.8, the model can achieve the optimal pruning effect. As shown in **Figure 12B**, a total of 89 network channels in YOLO-Ginseng were pruned, with a cumulative removal of 12,038 channels. This indicates that the channel pruning algorithm employed in this study effectively reduces the model parameter count.

To ensure that the pruned model maintains a high learning capability and detection performance, fine-tuning of the model is necessary. This is achieved by utilizing the pruned model as a pre-trained model for iterative training, thereby restoring the model’s detection performance. **Table 6** presents the variations in average precision, model parameter count, weight size, and inference time of YOLO-Ginseng throughout the entire model compression process. The results indicate that, following fine-tuning, the compressed YOLO-Ginseng model exhibits reductions of 65.3%, 76.4%, and 79.3% in inference time, weight size, and parameter count, respectively, compared to the initial model. Meanwhile, the average precision only experiences a marginal decrease of 0.2%.

**Table 6:** Comparison results of the model compression process.

Evaluation Standard	Initial model	Sparse training	Model pruning	Model fine-tuning
$\text{A}{\text{P}}_{0.5}$ **(%)**	**95.8**	**91.6**	**91.4**	**95.6**
**Parameters**	**21994034**	**21994034**	**4545903**	**4545903**
**Model size (MB)**	**44.5**	**44.5**	**13.4**	**10.5**
**Inference (ms)**	**21.3**	**17.8**	**8.4**	**7.4**

The bold values represent the best results in different evaluation indicators at different stages of model compression; the rows represent different stages of model compression, and the columns represent different evaluation indicators.

## 4 Discussion

### 4.1 The impact of data augmentation on model performance

In order to evaluate the impact of ginseng fruit image data augmentation on model performance, this study selected two groups of models, YOLOv5s and YOLO-Ginseng (uncompressed), for comparative experiments. The comparison results are shown in **Table 7**. Among them, data type A represents the original dataset without amplification, data type B represents the final dataset after amplification, that is, the ginseng fruit image dataset finally established in this study, $\Delta_{1}$ represents the average precision difference between the training set and the validation set, and $\Delta_{2}$ represents the total loss difference between the training set and the validation set. After 200 rounds of iterative training, the total loss of the training set and the validation set continued to decrease and gradually converged. In order to eliminate the impact of fluctuations in the early stage of training and focus on the performance of the model after stabilization, this study selected the average total loss value of the last 20 rounds for loss difference calculation. The results show that there is no significant change in the indicators of YOLOv5s before and after data augmentation; in YOLO-Ginseng, data augmentation improves the average accuracy of the model by 0.7%, and other evaluation criteria do not change much. For the average precision difference and the total loss difference, all difference results are small, indicating that the performance of the model in the training set and the validation set is relatively consistent, and both can maintain a low loss. This further shows that the model has good generalization ability on the two data sets A and B, and there is no over-reliance on the training set. All models perform normally on the two data sets A and B.

**Table 7:** Comparison results of data augmentation on model performance.

Type	Model	$AP_{0.5}$ (%)	T (ms)	Size (M)	Parameters	GFLOPS	$\Delta_{1}$	$\Delta_{2}$
**A**	**YOLOv5s**	**93.5**	**6.1**	**14.4**	**7012822**	**15.8**	**0**	**0.016**
**B**	**YOLOv5s**	**93.2**	**5.5**	**14.4**	**7022326**	**15.9**	**0**	**0.023**
**A**	**YOLO-Ginseng**	**95.1**	**21.1**	**42.4**	**21967879**	**35.8**	**0.1**	**0.017**
**B**	**YOLO-Ginseng**	**95.8**	**21.3**	**44.5**	**21994034**	**36.0**	**0**	**0.006**

The bold values indicate the best results of different models on different types of datasets A and B.

In summary, the ginseng fruit image dataset established in this study is reasonable, and data augmentation has made a certain contribution to the improvement of the average precision of YOLO-Ginseng.

### 4.2 Effect of different models on ginseng fruit detection

**Figure 13:**
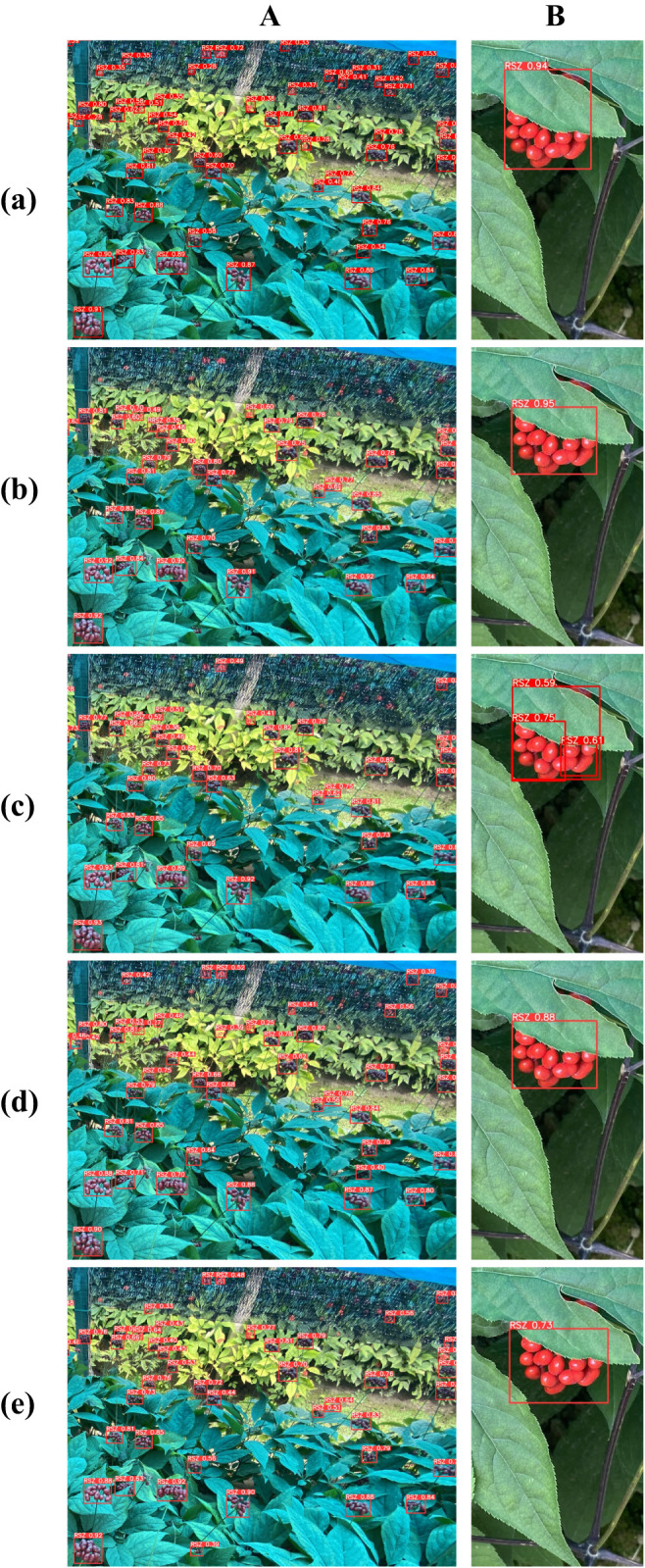
The performance of different models in detecting ginseng fruits in dense and complex scenes **(A)** and occluded scenes **(B)**. **(a)** YOLO-Ginseng. **(b)** YOLOv5s. **(c)** YOLOv7. **(d)** YOLOv8s. **(e)** YOLOv9-C.

To assess the effectiveness of YOLO-Ginseng in ginseng fruit detection, this section conducted comparative experiments by selecting seven different models for evaluation alongside YOLO-Ginseng. **Table 8** presents the comparative results of YOLOv3-tiny (Adarsh et al., 2020), YOLOv4-tiny (Bochkovskiy et al., 2020), YOLOv5s, YOLOv7, YOLOv7-tiny, YOLOv8s, YOLOv9-C, and YOLO-Ginseng. YOLOv3-tiny, YOLOv5s, YOLOv7, YOLOv8s, YOLOv9-C and YOLO-Ginseng were selected for P-R curve comparison. The comparison effect is shown in **Figure 9**. The results indicate that, compared to YOLOv5s, YOLO-Ginseng exhibits an improvement of 2.4% in average precision; The model inference time is increased by 1.9ms, finally reaching 7.4ms; It has the smallest model weight size, parameter count, and computational load. Compared to the remaining models, YOLO-Ginseng exhibits superior performance across all evaluation metrics. Among them, YOLO-Ginseng surpasses the relatively newer YOLOv7 and YOLOv8s and YOLOv9-C by 1.8% and 1.4% and 0.9% in terms of average precision; It is the fastest in terms of model inference time; It exhibits reductions of 86.0% and 50.9% and 89.8% in model weight size, 87.5% and 59.1% and 91.1% in parameter count, and 90.9% and 67.3% and 96.1% in computational load, respectively. **Figure 13** presents the detection performance of YOLO-Ginseng, YOLOv5s, YOLOv7, YOLOv8s, and YOLOv9-C in dense and complex scenes (A) and occluded scenes (B) for ginseng fruit detection. The results indicate that compared to YOLO-Ginseng, the other models exhibit more instances of missed detections when detecting ginseng fruit in dense and complex scenes, particularly in distant detection of ginseng fruit. However, although YOLO-Ginseng can detect most of the ginseng fruits globally, the detection effect of long-distance ginseng fruits is still not ideal, and its regression confidence is low. This may be because the long-distance ginseng fruits are not only small targets, Moreover, it was also caused by other ginseng fruits, leaves or other environmental factors blocking the detection. But overall, YOLO-Ginseng still has better detection results than other models. In the scenario of close-range occlusion of ginseng fruits, all models can detect the occluded ginseng fruits. However, in images (b), (d), and (e), the localization of the predicted bounding boxes is not precise enough, failing to fully capture all features of the occluded ginseng fruits. In image (c), multiple overlapping boxes are detected for the same ginseng fruit target, indicating occurrences of false positives. Additionally, in images (c), (d), and (e), the regression confidence for detecting occluded ginseng fruits at close range is low.

**Table 8:** Comparison results of different models.

Model	P (%)	R (%)	$AP_{0.5}$ (%)	T (ms)	Size (MB)	Parameters	GFLOPS
**Our method**	**93.6**	**91.1**	**95.6**	**7.4**	**10.5**	**4545903**	**9.3**
**YOLOv3-tiny**	**89.2**	**80.8**	**86.7**	**19.3**	**17.4**	**8666692**	**12.9**
**YOLOv4-tiny**	**89.5**	**85.8**	**89.2**	**8.9**	**20.6**	**7540110**	**13.6**
**YOLOv5s**	**90.9**	**87.0**	**93.2**	**5.5**	**14.4**	**7022326**	**15.9**
**YOLOv7**	**90.2**	**87.2**	**93.8**	**22.8**	**74.8**	**36481772**	**103.2**
**YOLOv7-tiny**	**90.9**	**87.5**	**93.7**	**11.4**	**23.2**	**6014988**	**13.2**
**YOLOv8s**	**90.7**	**88.2**	**94.2**	**14.2**	**21.4**	**11125971**	**28.4**
**YOLOv9-C**	**91.6**	**86.7**	**94.7**	**41.9**	**102.8**	**50958630**	**237.6**

The bold values indicate the best results of different models on different evaluation metrics.

In summary, YOLO-Ginseng has the best detection performance for ginseng fruits.

### 4.3 Comparison results of different models based on a public dataset

To further evaluate the detection performance of YOLO-Ginseng, this section will conduct tests using the public dataset PASCAL VOC2012. Comparative experiments will be carried out with YOLOv3-tiny, YOLOv4-tiny, YOLOv5s, YOLOv7, YOLOv7-tiny, YOLOv8s, and YOLOv9-C under the same PC device and training parameters. The comparative results are presented in **Table 9**. The results indicate that, on the public dataset, YOLO-Ginseng maintains superior performance across various evaluation standard, achieving an average precision of 70.8%, only below YOLOv7’s 76.7%; The model’s inference time reaches 6.8 ms, slightly inferior to other models. However, YOLO-Ginseng exhibits the smallest model weight size, parameter count, and computational load. In summary, YOLO-Ginseng demonstrates certain advantages in detection performance compared to other models on the public dataset PASCAL VOC2012.

**Table 9:** Comparison results based on the PASCAL VOC2012 dataset.

Model	P (%)	R (%)	$AP_{0.5}$ (%)	T (ms)	Size (MB)	Parameters	GFLOPS
**Our method**	**76.2**	**70.2**	**70.8**	**6.8**	**12.9**	**4572763**	**10.1**
**YOLOv3-tiny**	**50.3**	**46.7**	**43.4**	**3.5**	**17.5**	**8710582**	**13.0**
**YOLOv4-tiny**	**62.1**	**54.2**	**56.5**	**5.1**	**18.3**	**7340251**	**13.8**
**YOLOv5s**	**72.0**	**62.2**	**66.5**	**4.6**	**14.4**	**7064065**	**15.9**
**YOLOv7**	**75.2**	**71.9**	**76.7**	**13.1**	**75.0**	**37299042**	**105.4**
**YOLOv7-tiny**	**71.2**	**60.3**	**65.7**	**4.4**	**12.4**	**6059010**	**13.2**
**YOLOv8s**	**73.7**	**65.1**	**69.8**	**4.1**	**22.5**	**11133324**	**28.5**
**YOLOv9-C**	**69.6**	**63.6**	**68.8**	**14.6**	**102.9**	**50742168**	**236.9**

The bold values indicate the best results of different models on different evaluation metrics.

### 4.4 Deployment experiments of the model

**Figure 14:**
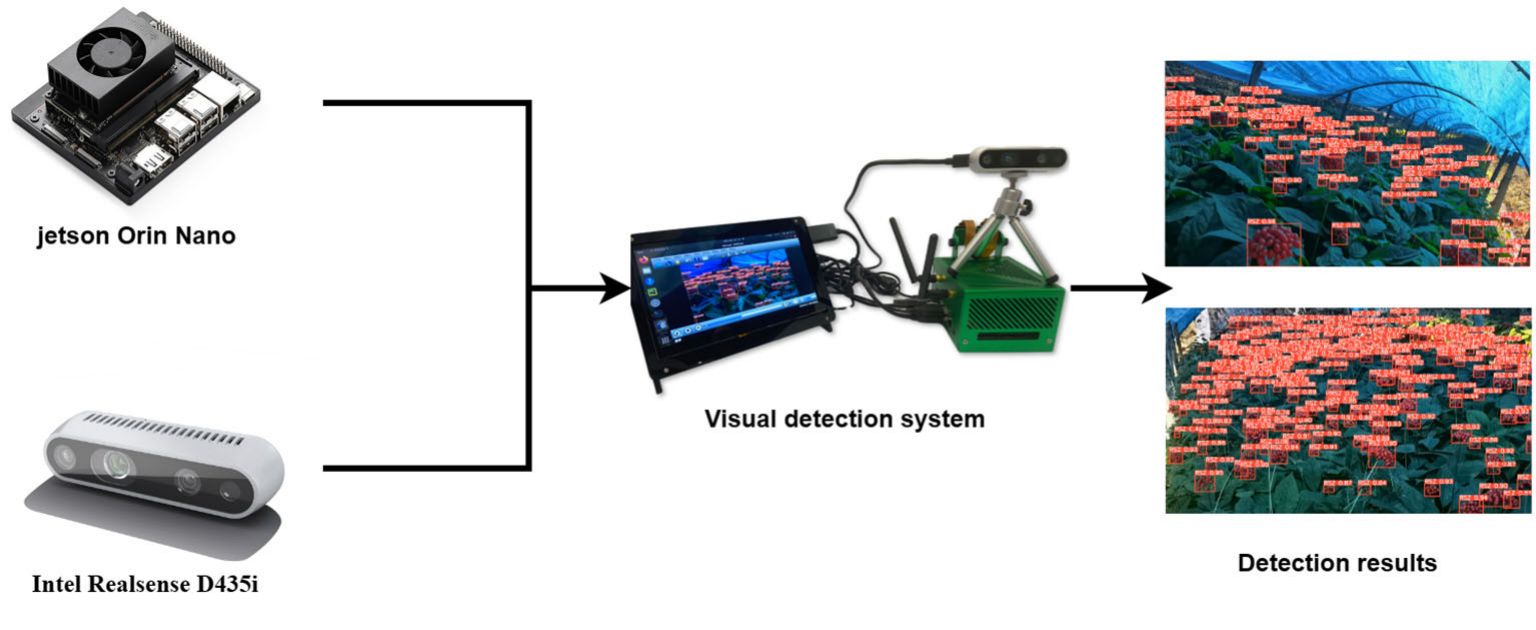
Model deployment platform and test results.

Finally, to validate the practicality and stability of YOLO-Ginseng, this study deployed the model on the Jetson Orin Nano computing device for simulated ginseng fruit detection experiments. The Jetson Orin Nano is equipped with 20 TOPS of computing power and supports a wide range of AI inference frameworks and tools. In this experiment, the RGB lens of the Intel Realsense D435i depth camera is used to capture image data, and the detection results were presented on a 7-inch touch screen. The visual detection system and detection results are depicted in **Figure 14**. The results demonstrate that YOLO-Ginseng can successfully accomplish ginseng fruit detection tasks on Jetson Orin Nano, achieving an average real-time detection speed of 24.9 fps. This indicates that YOLO-Ginseng possesses excellent practicality, meeting the detection requirements for future intelligent harvesting equipment of ginseng fruits.

## 5 Conclusions

This study proposes a ginseng fruit detection method, YOLO-Ginseng, which demonstrates outstanding overall detection performance and can provide visual guidance for ginseng fruit harvesting robots. The main contributions and conclusions of YOLO-Ginseng are as follows:

1. From the perspective of enhancing the feature extraction capabilities of the backbone network, this study designed a plug-and-play deep perception feature extraction module C3f-RN with a sliding window mechanism. This module expands the hierarchical structure of the YOLO-Ginseng backbone network, improves the backbone network’s local deep attention and global interactive processing capabilities for ginseng fruit image feature information, expands the network’s deep perception field of view, and can retain more important weight information. In the end, this method improved the localization quality of predicting boundary boxes for closely detected ginseng fruits, significantly reduced the missed detection rate of global ginseng fruit detection, enhanced the detection effectiveness of ginseng fruits at long distances, ultimately resulting in a 2.6% increase in the model’s average precision.
2. To mitigate the drawbacks caused by the C3f-RN module and maintain a balance between the detection precision and inference speed of YOLO-Ginseng, this study employed channel pruning algorithms for model compression. The results indicate that compared to the model before compression, YOLO-Ginseng experiences only a 0.2% decrease in average precision after compression. Meanwhile, the inference time, model weight size, parameter count, and computational load decrease by 65.3%, 76.4%, 79.3%, and 74.2%, respectively. This demonstrates the effectiveness of the channel pruning algorithm used for YOLO-Ginseng.
3. Finally, YOLO-Ginseng achieves a precision of 93.6%, a recall of 91.1%, an average precision of 95.6%, an inference time of 7.4ms, a model weight size of 10.5MB, 4,545,903 parameters, and a computational load of 9.3 GFLOPS, the detection effect is remarkable. It is noteworthy that YOLO-Ginseng exhibits the best overall detection performance compared to other models. On the publicly dataset, YOLO-Ginseng also demonstrates certain advantages in detection. In the model deployment, YOLO-Ginseng successfully accomplishes real-time detection tasks for ginseng fruits on the Jetson Orin Nano computing device, with an average detection speed reaching 24.9fps. However, YOLO-Ginseng has poor detection results for blocked ginseng fruits in long-distance detection scenarios, which is a problem that needs to be solved in subsequent research. In summary, this study provides effective visual guidance for ginseng fruit intelligent harvesting equipment, builds a bridge for ginseng fruit spatial positioning technology, and promotes the healthy development of the ginseng industry in the future.

## Data Availability

The raw data supporting the conclusions of this article will be made available by the authors, without undue reservation.
