# Supplementary figures and images for: YOLO-Ginseng: a detection method for ginseng fruit in natural agricultural environment
Source: Front Plant Sci. 2024 Nov 20;15:1422460. doi: 10.3389/fpls.2024.1422460 (PMC11618388; doi:10.3389/fpls.2024.1422460)

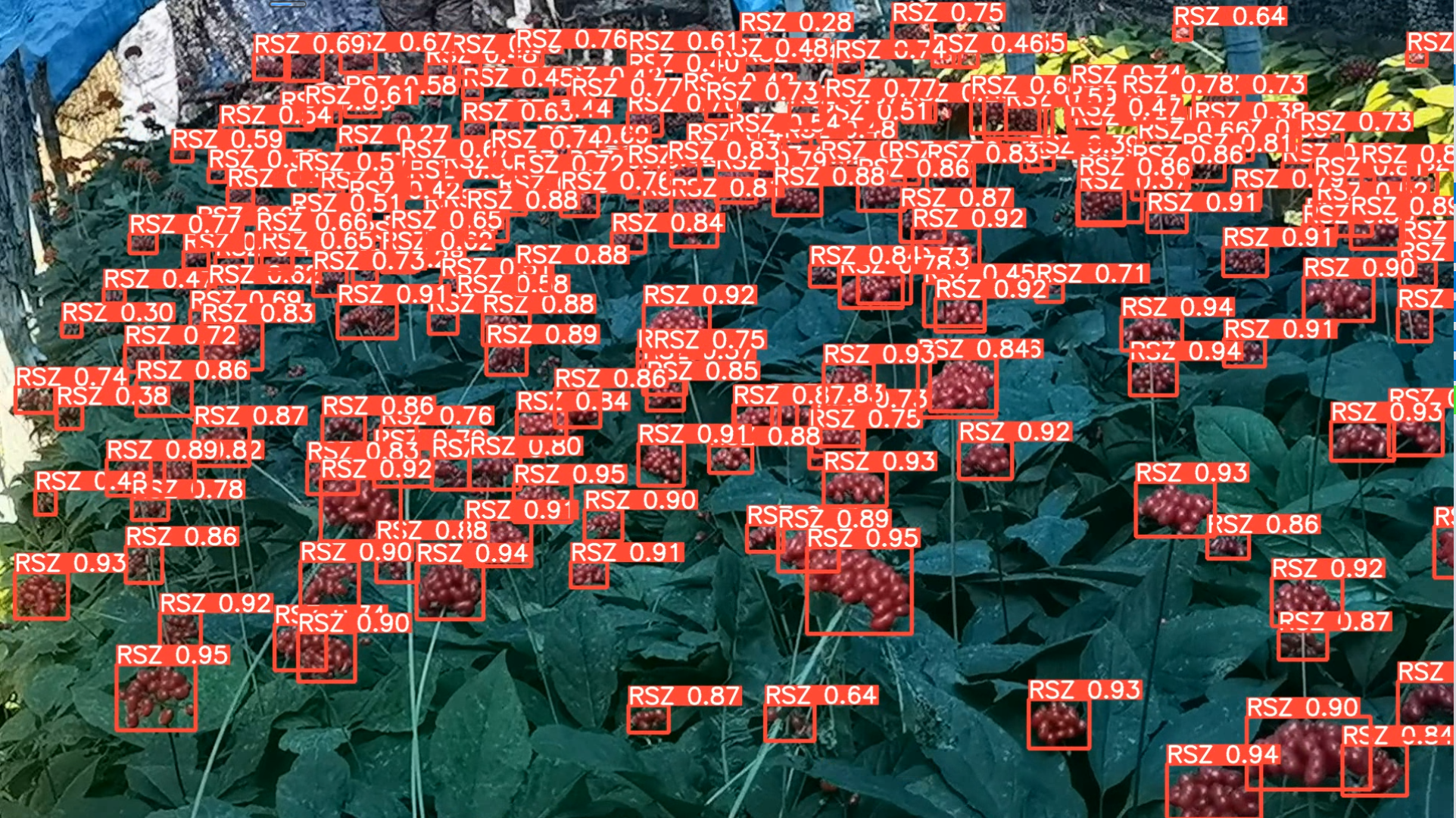

Supplement: Supplementary file 1 [file DataSheet1.zip › Supplementary_Material/Detection results after deployment on Jetson Orin Nano/2.png]

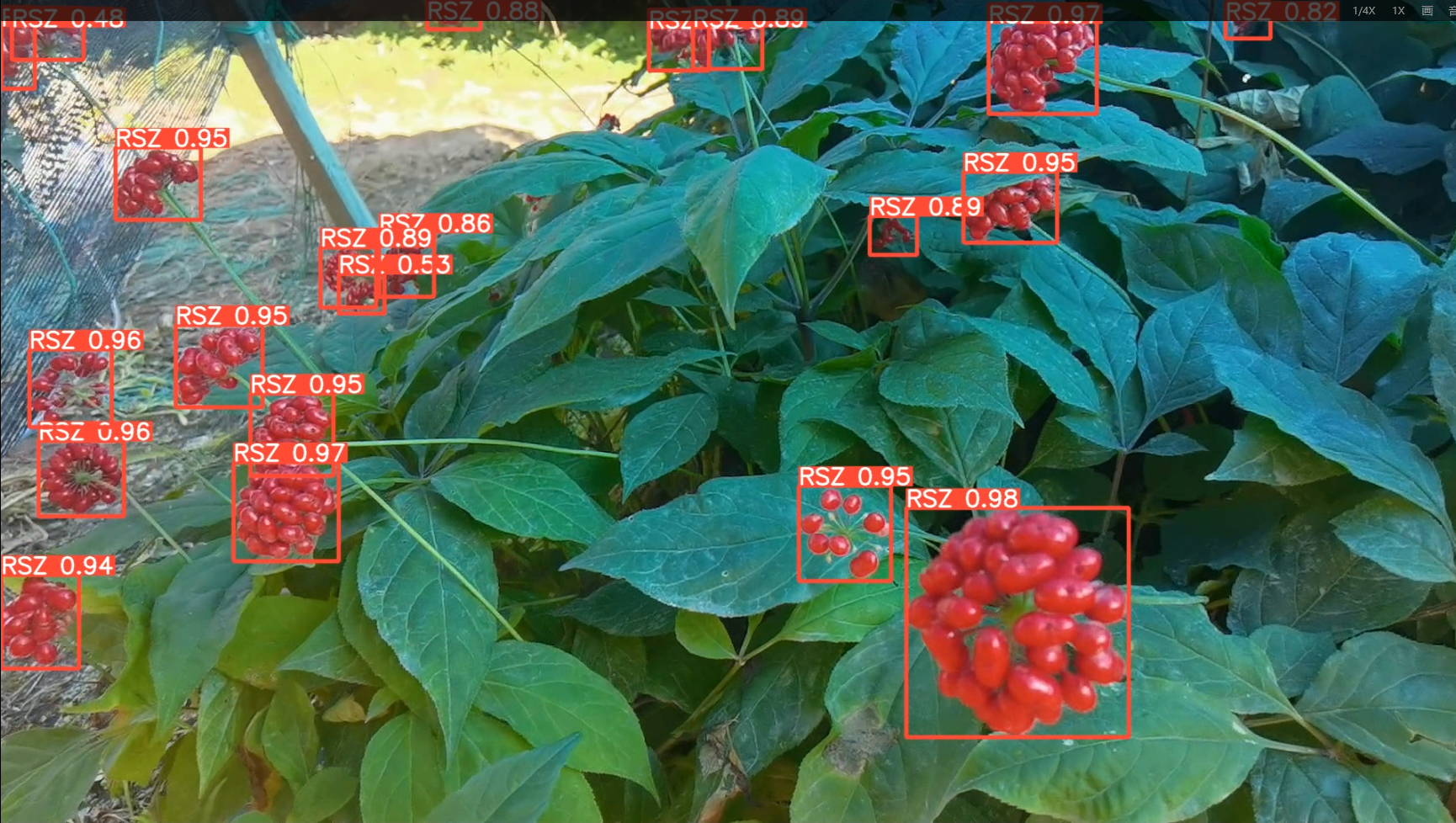

Supplement: Supplementary file 1 [file DataSheet1.zip › Supplementary_Material/Detection results after deployment on Jetson Orin Nano/3.png]

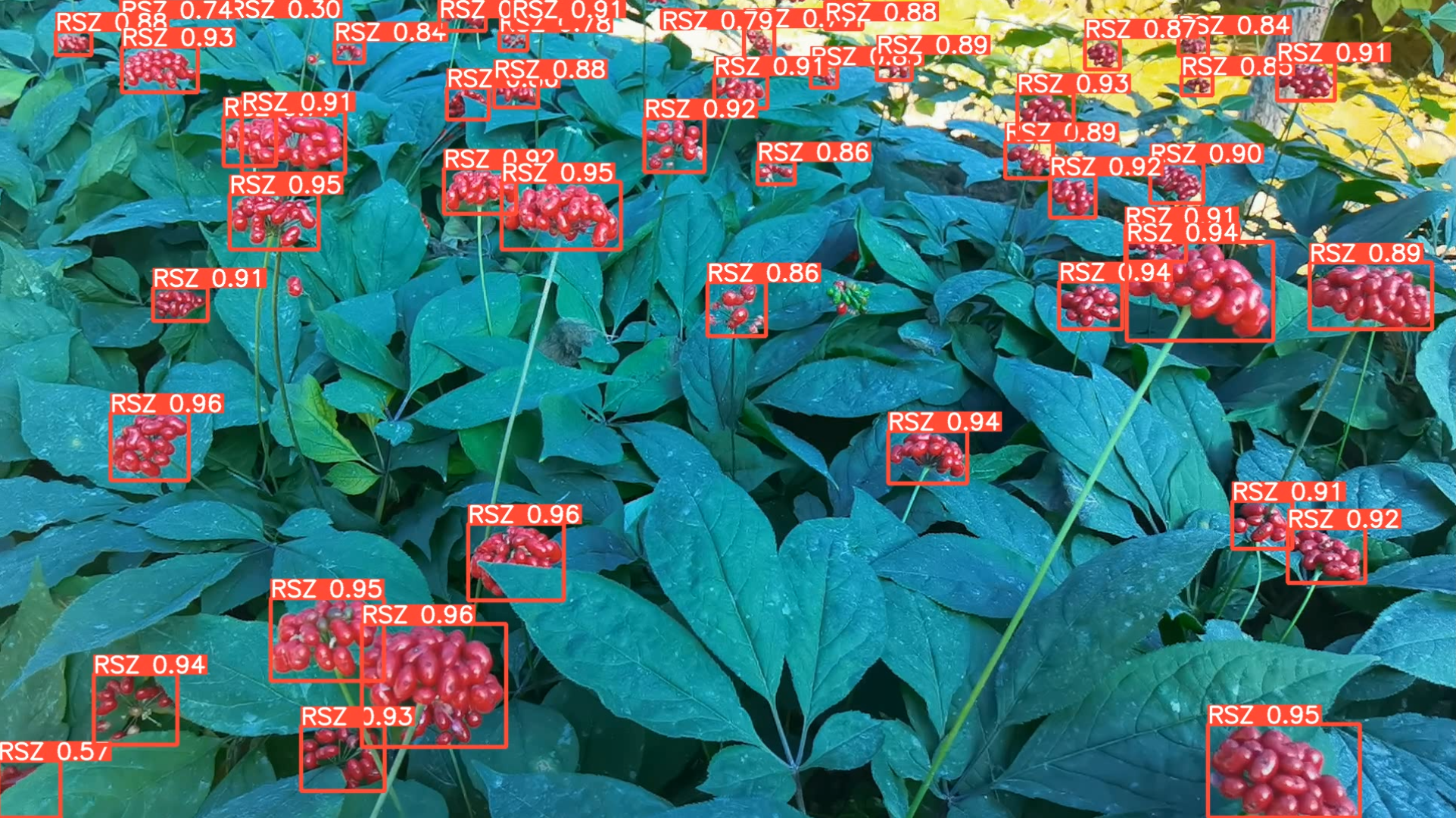

Supplement: Supplementary file 1 [file DataSheet1.zip › Supplementary_Material/Detection results after deployment on Jetson Orin Nano/4.png]

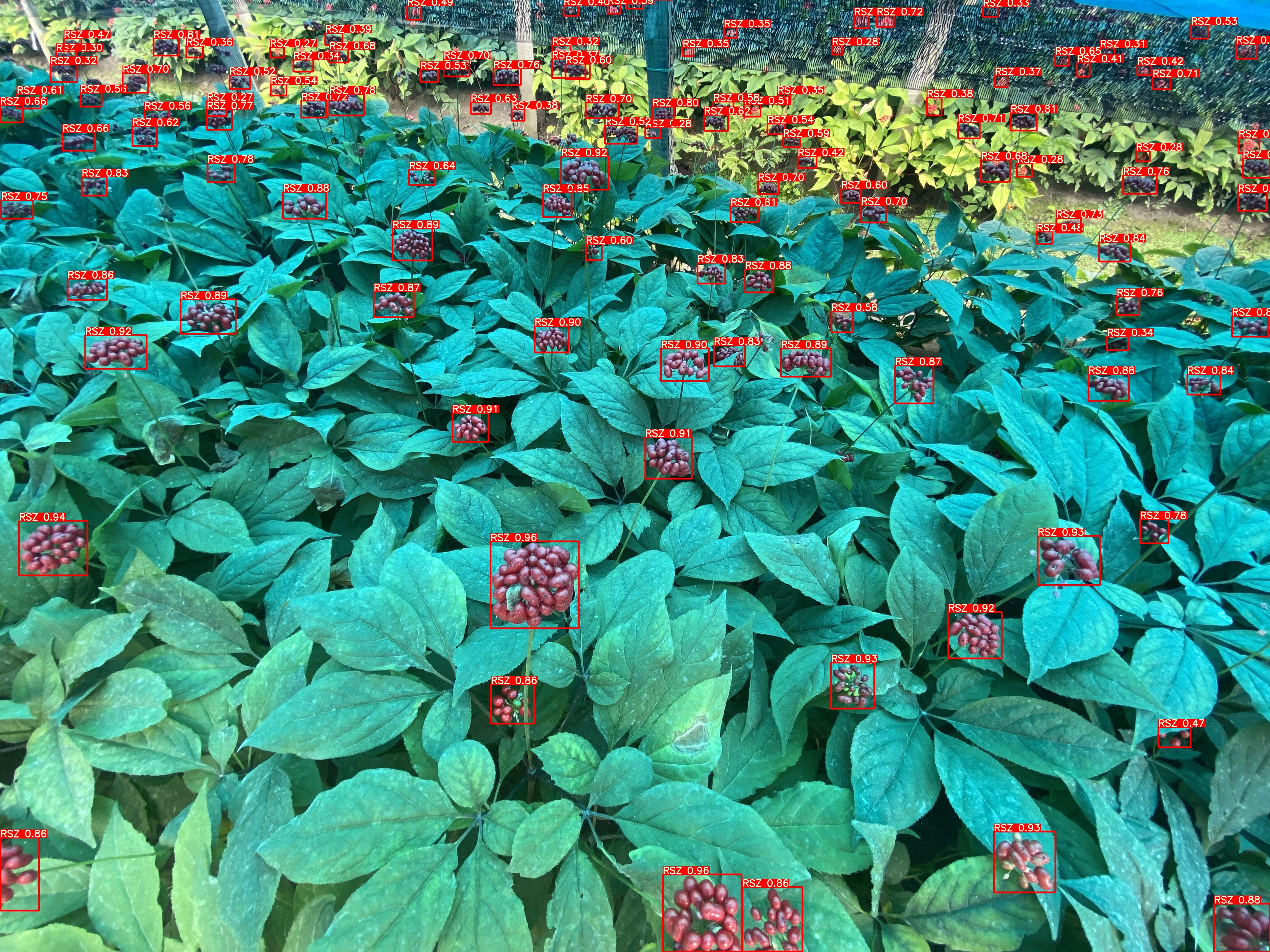

Supplement: Supplementary file 1 [file DataSheet1.zip › Supplementary_Material/Global detection results of image data in Figure 10/(a).JPG]

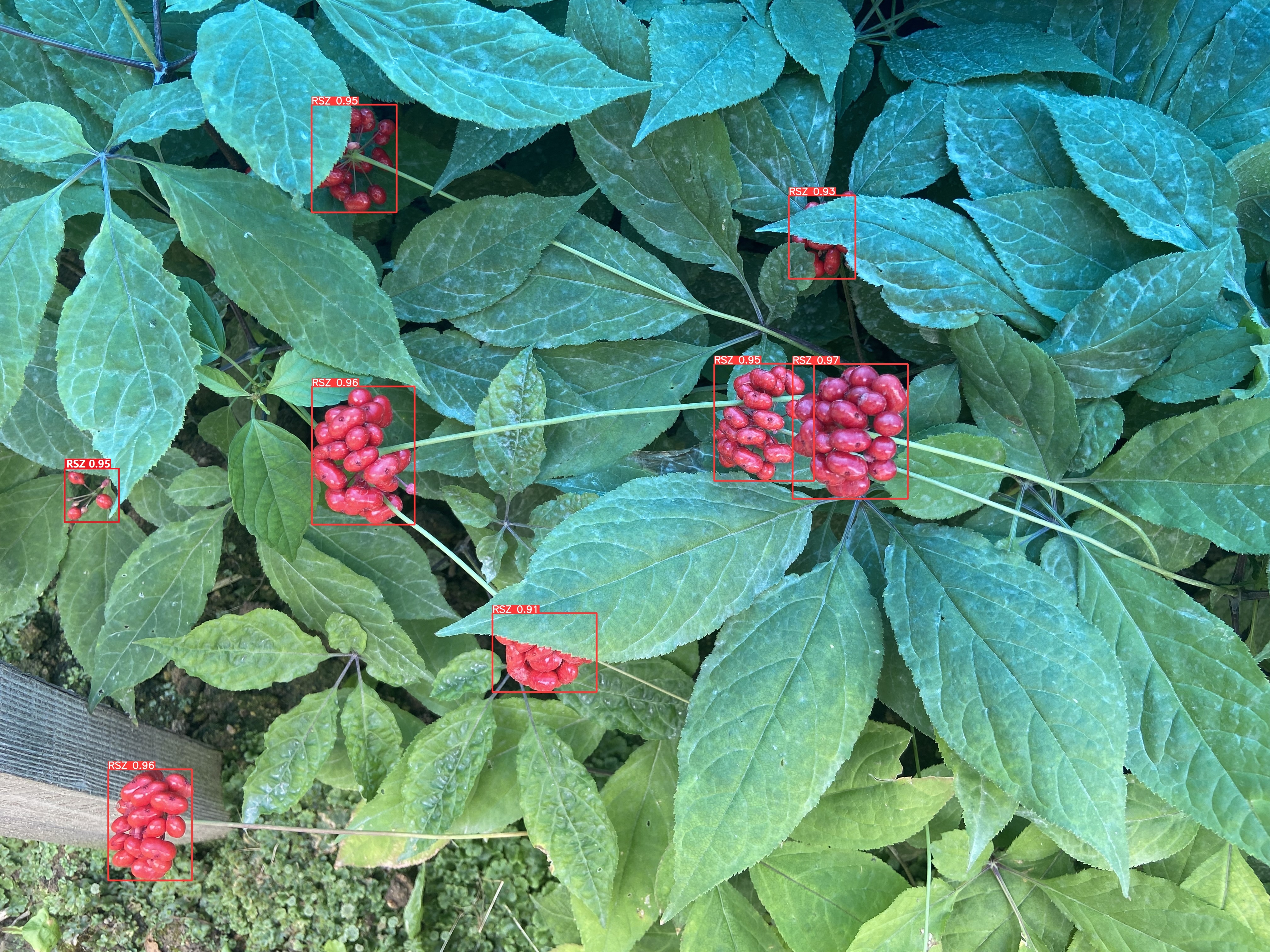

Supplement: Supplementary file 1 [file DataSheet1.zip › Supplementary_Material/Global detection results of image data in Figure 10/(b).JPG]
